# Cognitive Assessment in Adults With Adrenal Cortisol Insufficiency: Challenges and Opportunities

**DOI:** 10.1210/endrev/bnaf029

**Published:** 2025-09-03

**Authors:** Anat Ben-Shlomo, Michelle Koh, Sarah Kremen, Jeffrey Wertheimer

**Affiliations:** Department of Medicine, Multidisciplinary Adrenal Program, Pituitary Center, Cedars-Sinai Medical Center, Los Angeles, CA 90048, USA; Department of Medicine, Division of Endocrinology, Diabetes and Metabolism, Cedars-Sinai Medical Center, Los Angeles, CA 90048, USA; Department of Medicine, Division of Endocrinology, Diabetes and Metabolism, Cedars-Sinai Medical Center, Los Angeles, CA 90048, USA; Department of Neurology, Neurobehavior Program, Cedars-Sinai Medical Center, Los Angeles, CA 90048, USA; Department of Physical Medicine and Rehabilitation, Psychology and Neuropsychology Services, Cedars-Sinai Medical Center, Los Angeles, CA 90048, USA

**Keywords:** adrenal cortisol insufficiency, hydrocortisone, cognitive dysfunction

## Abstract

Adults with adrenal cortisol insufficiency (ACI) often report cognitive dysfunction, especially in memory processing and executive function. Only a few studies have objectively compared cognitive function as the primary outcome between patients with ACI and controls, and these efforts have yielded inconsistent results. In this review, we examine the challenges facing researchers studying cognitive function in adult patients with ACI. We consider the effect of dysregulated cortisol on cognition in patients with ACI, and the inability of current guideline-recommended glucocorticoid (GC) treatment regimens to accurately reproduce circadian and ultradian cortisol secretion rhythms. Factors that contribute to inter- and intra-individual response to GC are presented; the indirect effects of ACI comorbidities, complications, and symptoms on cognitive dysfunction are reviewed; and obstacles to employing neurocognitive testing are identified. Finally, we outline potential approaches to studying cognition in ACI using well-designed studies that account for the complexities and gaps in ACI research.

## Essential Points

- Cognitive dysfunction is commonly reported by adults with adrenal cortisol insufficiency (ACI)
- The few studies designed to objectively assess cognitive function in ACI compared to controls yielded inconsistent results
- Cortisol is a major regulator of cognitive function
- Current guideline-recommended glucocorticoid treatment regimens do not accurately reproduce circadian and ultradian cortisol secretion rhythms
- Cognitive function in ACI may be affected by symptoms of ACI such as fatigue, sleep disturbances, depression, and anxiety, as well as by comorbidities such as growth hormone deficiency
- Feasibility, accessibility, and psychometric quality of cognitive tests should guide instrument selection within the individual clinical context

Adrenal cortisol insufficiency (ACI) is a rare, life-threatening condition in which the adrenal glands fail to produce sufficient cortisol to maintain metabolic homeostasis and adequately respond to stress (1-3). Despite treatment with glucocorticoids (GC) as cortisol replacement, patients with ACI consistently report cognitive challenges (1, 4-9). However, few studies have used objective assessments of cognitive function to quantify impairment compared to matched controls, and these efforts have yielded inconsistent results (5, 6, 10-12). Consequently, this clinically significant symptom remains largely unaddressed in the care of patients with ACI.

**Figure 1.**
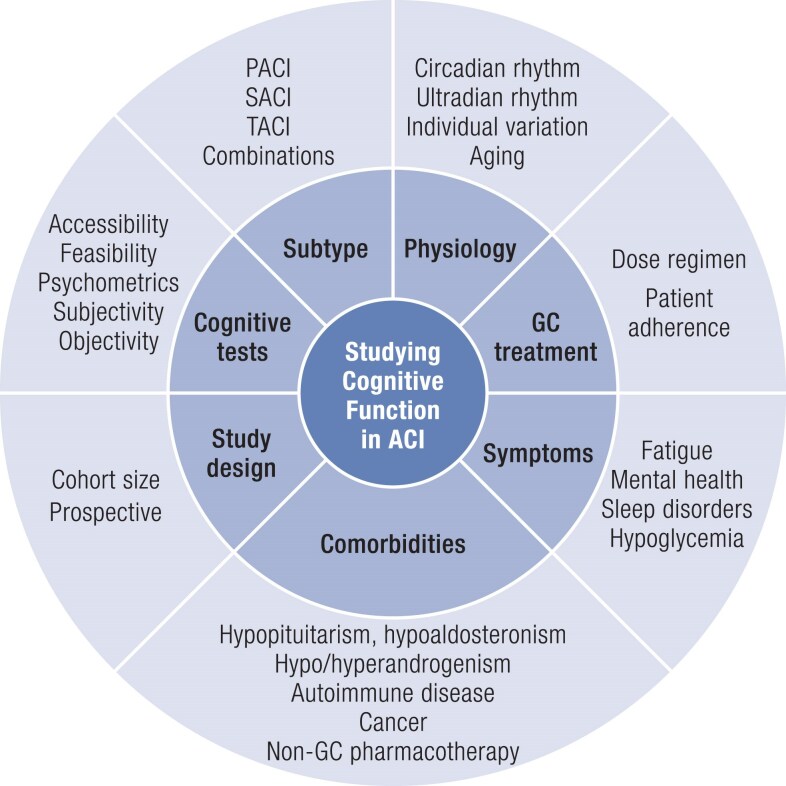
Considerations in studying cognitive function in ACI. Representation of the multiple factors that can directly and indirectly affect cognition in patients with ACI, including disease subtype, physiological disruptions, GC treatment, and disease symptoms and comorbidities, as well as factors such as study design and test selection that can influence accurate assessment of cognitive function.

In this review, as a first step in addressing these diagnostic and treatment gaps, we examine the challenges facing researchers studying cognitive function in patients with ACI, focusing specifically on adults. We briefly explore the relationship between circulating cortisol levels and cognition, discuss why application of current treatment guidelines (13, 14) complicates accurate measures of cognitive function in ACI, and consider the indirect effect of ACI-related comorbidities and symptoms on cognition (Fig. 1). We also discuss how methodological flaws can compromise accuracy of study results. We propose a cross-disciplinary, integrated team approach that fosters rigorous study of cognitive function in patients with ACI, and which, in turn, will provide reliable insights that can be applied in clinical practice.

Throughout this review, we use the term *adrenal cortisol insufficiency* rather than *adrenal insufficiency* or *hypoadrenalism* to focus on the specific effects of cortisol on cognitive function. With this usage we are not proposing a new terminology and are not using the term and the acronym with the intent that they be adopted formally in the field. However, as we discuss below, current treatment with GC does not accurately reproduce endogenous cortisol secretion rhythms (13, 14) and lessons learned from the effects of excess cortisol (7, 15) suggest that inadequate cortisol replacement may drive some of the cognitive deficits reported by patients with ACI. Thus, this term is particularly useful for this manuscript.

## Overview of Adrenal Cortisol Insufficiency

Patients with ACI have one or more points of damage along the hypothalamic-pituitary-adrenal (HPA) axis that result in cortisol deficiency (16). Primary ACI (PACI) is caused by adrenal gland disorders and often results in deficiency of all adrenal steroids, including cortisol, aldosterone, and androgens. Autoimmune destruction, or Addison disease, is the most common cause of PACI in the Western world, followed by infectious destruction, bilateral adrenalectomy, and inborn mutations (17). Prevalence of PACI has been estimated at 82 to 144 cases per million persons, with the incidence of autoimmune PACI (4.4-6.0 per million persons per year) peaking at ages 30 to 50, with 60% of cases in female individuals (18). Cortisol deficiency in patients with PACI typically results in a compensatory increase in pituitary adrenocorticotropic hormone (ACTH) secretion (14); patients with PACI due to autoimmune adrenalitis often also have hypoaldosteronism, hypoandrogenism (particularly in female patients), or hyperandrogenism (particularly in patients with select steroidogenesis enzyme mutations) (19). Many patients will also have Hashimoto autoimmune thyroiditis, and rarely other endocrine gland and nonendocrine autoimmune disorders as part of the autoimmune polyglandular syndrome (14).

Patients with central ACI are classified as having either secondary ACI (SACI) or tertiary ACI (TACI) depending on the tier of HPA axis disruption (16). SACI results from a pituitary disorder, leading to decreased ACTH production and therefore decreased adrenal cortisol production. The most common cause is a sellar tumor (17). Other pituitary insults from radiation, hemorrhage, and inflammation can also lead to SACI (13). Prevalence of SACI has been estimated at 150 to 280 cases per million persons (18). Lymphocytic autoimmune hypophysitis resulting from the use of novel immune checkpoint inhibitors in the treatment of cancer (20, 21) may have increased the prevalence of SACI over the last decade. SACI tends to be seen more frequently in patients ages 50 to 60 due to the increased prevalence of most types of pituitary adenomas with age (22). Comorbidities associated with other axes originating in the pituitary gland may be present, such as hypogonadism, growth hormone (GH) deficiency, hypothyroidism, hyper- or hypoprolactinemia, and occasionally vasopressin deficiency (13).

TACI results from a hypothalamic disorder, which inhibits production and/or action of corticotropic releasing hormone (CRH) and consequently reduces both ACTH and cortisol secretion. Chronic TACI may be seen in patients with hypothalamic tumors such as craniopharyngioma (23). Most commonly, TACI is transient and results from GC medication use that suppresses the HPA axis, decreasing hypothalamic CRH and pituitary ACTH production (1). In these patients, normal HPA axis functioning typically recovers and symptoms resolve after GC tapering and withdrawal (24).

Regardless of the etiology of central ACI, as ACTH is not the major regulator of adrenal aldosterone, these patients typically do not experience hypoaldosteronism or require mineralocorticoid replacement (13).

## Cognitive Dysfunction and Dysregulated Cortisol

Cortisol affects central nervous system development, metabolism, and function (25). Lipophilic cortisol, which is readily transferred from the circulation, is the major source of brain cortisol (26, 27). Cortisol can also be produced locally from conversion of cortisone to cortisol by the 11β-hydroxysteroid dehydrogenase type 1 (11β-HSD1) enzyme (7, 28).

In the human brain, cortisol binds both GC receptors (GR) and mineralocorticoid receptors (MR). mRNA expression in postmortem studies of healthy human donors shows GR gene (*NR3C1*) ubiquitously expressed throughout the brain while MR gene (*NR3C2*) distribution is largely limited to the limbic system (29-32).

### Lessons Learned From Cognitive Dysfunction in Chronic Excess Cortisol States

Knowledge of cortisol's effect on cognition in patients with higher levels of circulating cortisol (7, 15, 33-36) can be informative for understanding its effects on cognition in patients with ACI. There are many studies associating higher cortisol levels with cognitive dysfunction. In subjects enrolled in the Framingham Heart Study, higher fasting morning cortisol levels was associated with worse memory and visual perception, even when levels were still within the normal range. Higher cortisol level was also associated with lower total cerebral brain volume in women, as well as lower occipital and frontal lobar gray matter volumes and multiple areas of microstructural changes in both sexes (36).

Furthermore, a slight chronic increase in endogenous cortisol levels above the upper limit of normal has been linked to mild cognitive impairment. One study of patients with adrenal incidentaloma and mild autonomous cortisol secretion (MACS) reported memory deficits at higher rates than in patients with nonfunctioning adrenal adenomas. Their poorer performance in visuospatial, constructional, and working memory domains on objective assessments was noted even after adjusting for age, sex, and years of education (37). In another study, objective assessment of cognitive function using the well-validated National Institutes of Health Toolbox–Cognition Battery showed worse scores for fluid, crystallized, and composite cognitive tests compared with the normal reference population, even after adjusting for age, sex, frailty, depression, and total GC dose (38).

There are also multiple studies showing compromised cognitive function and changes in brain structures in patients with Cushing syndrome (CS). A meta-analysis of 18 studies on cognitive deficits among individuals with active CS found significant impairments across multiple cognitive domains, including attention, processing speed, visuospatial perception, verbal learning and memory, language skills, and visual learning and memory, as well as overall intelligence, compared to healthy controls. Among those with ACTH-secreting pituitary adenomas (Cushing disease), postsurgical evaluations showed improved cognitive function, particularly in verbal and visual learning and memory, intelligence, and executive functions, irrespective of the time since the surgical intervention (39).

Finally, a systematic review of 40 studies evaluating cognitive function in patients with CS suggested that both higher cortisol levels and longer duration of uncontrolled disease were associated with more severe impairment (15). Importantly, in this systematic review, duration of remission was not associated with memory improvement, suggesting effects of long-term GC exposure on brain function may be irreversible (15). These findings are consistent with a separate systematic review of 19 studies evaluating patients with active CS using magnetic resonance imaging (MRI) or proton magnetic resonance spectroscopy, which demonstrated smaller hippocampi, larger ventricles, and cerebral atrophy along with changes in functional brain activity that only partially improved after correcting the underlying hypercortisolism (40). Although mechanisms linking structural and functional changes in brain imaging with cognitive dysfunction and excess cortisol levels remain unclear (41), functional connectivity aberrations in key brain networks, including the limbic network (amygdala, hippocampus, and cingulate gyrus) responsible for memory and emotional and stress responses; the default mode network (medial prefrontal cortex, posterior cingulate cortex, and parietal regions) crucial for memory and conceptual processing; and the executive control network (frontoparietal regions) important for working memory and executive function have all been implicated in cognitive impairment (42, 43).

Thus, given that chronic excess of cortisol is detrimental to cognitive performance and brain structure, it seems likely that chronically low levels of cortisol in patients with ACI would also be detrimental to cognitive function. Indeed, an inverted U-shaped relationship has been suggested between cortisol levels and cognitive function, indicating that both high and low levels of GC exposure are detrimental to memory (44, 45).

### Cognitive Dysfunction in ACI

In contrast to the numerous studies examining the deleterious effect of excess cortisol on the brain, effects of cortisol deficiency are poorly studied (25).

The few studies conducted in patients with ACI show marked discrepancies in rates and severity of cognitive impairment between subjective self-reports and objective assessments, and most studies have focused nearly exclusively on PACI. Thus, interpretating data on cognitive dysfunction in ACI remains challenging.

On subjective questionnaires, patients with ACI report significant cognitive impairment. For example, markedly higher rates of difficulty with attention (57% vs 19%), memory (70% vs 26%), and executive functioning (25% vs 3%) were reported in one study of 60 patients with PACI compared with 30 sex- and education-matched controls (5). Another study found more self-reported executive dysfunction, self-organization difficulties, and abnormal emotional regulation such as anxiety in 39 female patients with ACI compared with 43 controls, even after excluding those with depression (6). Increased clumsiness and forgetfulness were reported in 60 patients with PACI compared to 60 age-, sex- and ethnicity-matched controls (4), and more self-reported difficulties concentrating were seen in 30 patients with PACI compared to 30 age-, sex-, and education-matched controls (11).

Only a handful of studies objectively compared cognitive function as the primary outcome between patients with ACI and controls (Table 1) (5, 6, 10-12). In contrast to the frequent impairments reported by patients with ACI on subjective questionnaires, objective assessments generally reveal only mild cognitive deficits or inconsistent results (8, 9).

**Table 1.** Summary of studies designed to evaluate cognitive function in adult patients with ACI vs matched controls

Study, Country	Subjects, ACI subtype, sex	Controls, matching	Age, mean (SD) [range] years	Disease/treatment duration, mean (SD) [range] years	Daily dose, mean (SD) [range]	Number of daily doses, mean (SD) [range]*^a^*	Objective cognitive tests administered	Key positive findings
Henry 2014 (10) South Africa	*n* = 27 PACI (20F, 7M)	*n* = 27 Matched for age, sex, education, race	*Subjects:* 48.70 (15.36) [20-72] *Controls:* 49.04 (15.11) [21-74]	15.36 (10.24) [1-50]	*HC (n* = *21):* 22.38 (6.82) [10-35] mg 0.33 (0.14) [0.13-0.70] mg/kg *Prednisone (n* = *3):* 16.67 (12.58) mg 0.12 (0.23) mg/kg	1.96 (0.68) [1-3] (*n* = 25)	Brief Test of Adult Cognition by Telephone, including: *Episodic memory:* Rey Auditory-Verbal Learning *Working memory:* Digit Span-Backwards *Executive function:* Category Fluency *Attention-switching reaction time* Red-Green (score) *Reasoning:* Number Series *Processing speed:* Counting Backwards	*Performance in subjects vs controls:* Worse episodic memory (immediate-delayed recall) *Predictors:* Correlation between disease duration and all tests except immediate-delayed recall and delayed false reports
Schultebraucks 2015 (11) Germany	*n* = 30 PACI (21F, 9M)	*n* = 30 Matched for age, sex, education, menstrual cycle phase (follicular vs luteal), menopausal status	*Subjects:* 52.4 (14.4) *Controls:* 52.0 (13.8)	18.2 (11.1) [4-53]	*HC (n* = *22)* and *DR-HC^b^ (n* = *8):* 21 (4.0) [15-35] mg 0.3 (0.1) [0.19-0.51] mg/kg	*HC (n* = *22):* 2.3 (0.5) [2-3] *DR-HC (n* = *8)*: 1	*Verbal learning and memory:* Auditory-Verbal Learning *Visual-spatial memory:* Rey-Osterrieth Complex Figure *Autobiographical Memory* Autobiographical memory test *Working memory:* Digit Span Task *Executive function:* Stroop test *Attention:* Number-Combination	*Performance in subjects vs controls:* Worse verbal learning *Predictors:* Correlation between SBP and verbal memory, visual-spatial memory Correlation between BMI and executive function
Tiemensma 2016 (5) The Netherlands	*n* = 31 PACI tested after morning HC (20F, 11M) Diagnosis: 19 AA, 2 non-AA, 1 bADX, 9 unknown *n* = 29 PACI tested before morning HC (17F, 12M) Diagnosis: 19 AA, 3 non-AA, 1 bADX, 6 unknown	*n* = 31 (20F, 11M) Matched for age, sex, education to 31 subjects tested after morning HC	*All subjects and controls:* 52.4 (14.4) *Subjects tested after HC:* 49 (11) *Subjects tested before HC:* 50 (14) *Controls:* 45 (12)	*All subjects:* 9.7 (8.2) [2-38] *Subjects tested after HC:* 10 (8) *Subjects tested before HC:* 10 (8)	*All subjects:* 0.31 mg/kg 12.2 mg/m^2^ *Subjects tested after HC:* 23 (7) mg 0.28 mg/kg 11.4 mg/m^2^ *Subjects tested before HC:* 26 (8) mg 0.34 mg/kg 13.3 mg/m^2^	NA	*Memory:* Wechsler Memory Scale Rey Verbal Learning Rey Complex Figure *Executive function:* Verbal Fluency Letter-Digit Substitution Stroop Interference Trail Making Sustained Attention to Response *Verbal intelligence:* Groninger Intelligence (GIT-2)	*Performance in subjects tested after HC vs controls:* Worse auditory and visual memory (worse logical memory, fewer words recalled, worse immediate and delayed recall for words and figures) but better concentration Worse executive function (more repeated words in verbal fluency, more time to complete number-letter trails), but better attention tasks (fewer errors in number-number trails *Performance in subjects tested before HC vs subjects tested after HC:* No change
Blacha 2021 (12) Germany	*n* = 21 PACI (16F, 5M) Diagnosis: 19 AA, 2 bADX *n* = 19 SACI (12F, 7M) Diagnosis: 14 postsurgery for sellar tumors (12 NFPA, 1 CP, 1 acromegaly), 2 Sheehan syndrome, 2 hypophysitis, 1 empty sella	*n* = 20 (11F, 9M) Matched to a PACI or SACI subject for age, sex, education	*All subjects:* 52.7 *Controls:* 51.6	*All subjects:* 10.7 [0-56]	*All subjects:* 25.8 [8-41] mg	2.5 (0.50) [1-3]	*Attention and visuomotor skills:* Digit Symbol Test *Memory:* Wechsler Adult Intelligence Scale *Executive function:* Trail Making Test *Divided attention:* d2-R Letter Cancellation test	*Performance in subjects vs controls:* Worse attention (prolonged reaction time, lower response to visual stimuli with no acoustic warning) *Predictors:* Worse time-based attention, executive function, and divided attention with HC ≥25 mg/d
van’t Westeinde 2022 (6) Sweden	*n* = 67 PACI (39F, 28M) Diagnosis: All AA	*n* = 80 (43F, 37M)	*Subjects:* 32.3 (6.7) [19-41.9] *Controls:* 29.2 (7.4) [19.0-43.4] (*P* = 0.010)	*All subjects:* 9.5 (4.8) [2.5-24.9]	*HC (n* = *52):* 13.1 (3.5) mg/m^2^ *DR-HC^b^ (n* = *15):* 11.8 (2.7) mg/m^2^	*HC:* 2.6 (0.7) [NA] *DR -HC:* 1.3 (0.6) [NA]	*Intellectual ability:* Wechsler Adult Intelligence Scale (WAIS) IV Matrices, Vocabulary *Executive function:* WAIS-IV Digit Span, Coding Wechsler Memory Scale (WMS) III Span Board*Processing speed*: WMS-III Word, Coding Stroop Interference *Learning and memory:* WMS-III Word List Learning Test	*Performance in subjects vs controls:* Lower verbal intellectual ability scores Lower visuospatial working memory scores Results were within average range of normal population *Predictors:* Longer time since last medication associated with better visuospatial working memory Executive functions were associated with mental fatigue and lower GC daily dose

Abbreviations: AA, autoimmune adrenalitis; ACI, adrenal cortisol insufficiency; bADX, bilateral adrenalectomy; BMI, body mass index; CAH, congenital adrenal hyperplasia; CP, craniopharyngioma; DR, dual-release; F, female; HC, hydrocortisone; M, male; NA, not available; NFPA, nonfunctioning pituitary adenoma; PACI, primary adrenal cortisol insufficiency; SACI, secondary adrenal cortisol insufficiency; SBP, systolic blood pressure.

^*a*^Number of daily doses is reported as provided in each study.

^*b*^Patients were treated with once-daily dual-release HC (Plenadren®), which is described in the study as modified-release or extended-release HC.

A unified interpretation of these objective findings is challenging. Nevertheless, several of the affected cognitive domains in patients with ACI—including episodic memory, verbal learning, and memory for verbally and visually mediated information—are associated with hippocampal function. Other identified deficits, particularly in executive function, attention, and verbal intellectual ability, are mediated by the prefrontal cortex. Both of these brain areas have been shown to express GR in humans (29, 31, 46, 47).

The authors of the 5 studies detailed in Table 1 acknowledge several key limitations, which have been further highlighted in 2 recent reviews (8, 9). Specifically, given the rarity of ACI, study cohorts are typically small, making it difficult to control for epidemiological, anthropometric, comorbidities, and treatment-related factors known to affect cognition. Furthermore, methodological limitations in study design and assessment tools make it difficult to distinguish subtle changes. As discussed in the sections that follow, identifying and addressing these challenges through improved study design and analysis will enhance our ability to accurately characterize and manage cognitive dysfunction in ACI.

## Potential Challenges to Address When Designing Studies Assessing Cognitive Function in ACI

### Effect of GC Replacement on Cognition in ACI

#### Circadian and ultradian rhythms

The HPA axis is a biological system in a “continuous dynamic equilibration” (48) that uses carefully orchestrated rhythms to regulate hormone levels. Briefly, the circadian rhythm is driven by external cues and internal biological clocks regulating production and secretion of hypothalamic CRH, pituitary ACTH, and adrenal cortisol. In general, in individuals without ACI, cortisol levels begin to rise between 02:00 and 03:00, and then gradually increase to reach a peak upon awakening. Cortisol levels gradually decrease over the course of the day, reach their nadir at night, and remain at the nadir level for the first half of the night until the circadian cycle restarts (49-51).

Superimposed on the circadian rhythm is the ultradian rhythm, an oscillatory pattern consisting of multiple small fluctuations in cortisol levels. This rhythm allows for tissue-specific, rhythmic GR responsiveness with rapid termination of action, facilitating precise, ligand-dependent gene expression pulsing (49, 50).

Disruptions in circadian rhythm and cortisol regulation have been associated with cognitive dysfunction (52). For example, attenuated cortisol daily amplitude and increased nadir cortisol levels seen with changes in sleep time and light exposure, such as seen in night shift conditions, resulted in reduced alertness and cognitive performance (53). The cortisol awakening response (CAR), an acute and timed peak in the cortisol level that occurs within the first 30 to 45 minutes after awakening, facilitates daytime neurocognitive processing, and a greater CAR may enhance cognition (54). By contrast, suppression of CAR with metyrapone, which inhibits conversion of 11-deoxycortisol to cortisol, leads to impaired memory retrieval 30 minutes after awakening (55).

The ultradian rhythm, which is absent in patients with ACI, has also been implicated in supporting cognitive function and its role in this setting is increasingly being recognized (52). For example, in healthy males with metyrapone-induced ACI, 5 days of continuous subcutaneous hydrocortisone (HC) infusion simulating both circadian and ultradian rhythms improved working memory during increasingly complex cognitive tasks, whereas treatment to simulate circadian rhythm alone did not (56). Yet, simulation of both rhythms in 22 patients with PACI enrolled in a 6-week randomized, crossover, double-blind, placebo-controlled study did not result in improved performance on tests of emotional processing, even though mood, fatigue, and perceived sleep quality all improved (57). It is possible that the very limited evaluation of cognitive function in this study resulted in missed opportunities to observe improvements, and that improvement might be demonstrated with a larger sample size and with assessment of other cognitive domains, including executive function, working or episodic memory, and verbal or visual learning. It is also possible that duration of ACI influenced outcomes, as effects of cortisol deficiency induced by short-term metyrapone treatment in healthy subjects may not be comparable to prolonged deficiency in patients with PACI. While some have reported a positive correlation between ACI duration and episodic memory (10), others have found no such association (12, 58). After adjusting for these and other confounders, simulation of both circadian and ultradian rhythm through continuous HC dosing may yet prove useful in this setting.

#### Dissonant circulating cortisol oscillations

**Figure 2.**
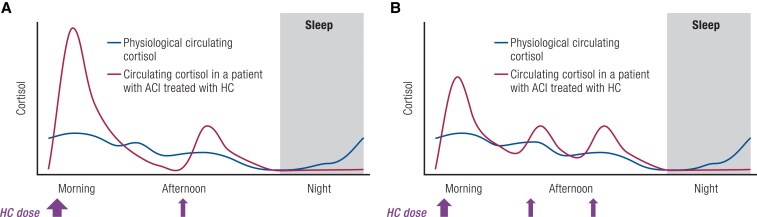
Dissonant circulating cortisol oscillations (DiCO) in ACI. Cartoon of physiological circulating cortisol level in individuals without ACI and measured circulating cortisol in a representative patient with ACI treated with exogenous HC when administered either (A) 2 times daily or (B) 3 times daily. Circulating levels of cortisol with HC treatment do not reproduce the cortisol circadian rhythm, resulting in periods of cortisol excess or deficiency throughout the day compared with expected physiological levels (59). Shaded area indicates sleep, when endogenous cortisol slowly rises from nadir levels in individuals without ACI but remains at nadir levels in patients with ACI.

Current management guidelines for patients with ACI recommend use of oral HC as GC replacement. A higher dose of oral HC is taken in the morning, followed by 1 to 2 additional lower doses thereafter, with the last dose taken in the afternoon (13, 14). This regimen is designed to, but does not, accurately reproduce circadian rhythm or at all replicate ultradian rhythm and CAR. Rather, it creates dissonant circulating cortisol oscillations (DiCO) that are misaligned with the physiological circulating cortisol rhythm observed in individuals without ACI. Patients display abnormal cortisol peaks and troughs during the day, as well as an absence of cortisol increase in the second half of the night and upon natural awakening (59) (Fig. 2).

Unfortunately, studies of cognitive function in ACI are all conducted in this artificial state of DiCO. Evaluating the true effects of the prolonged hypocortisolism of ACI on cognition would require discontinuing HC treatment for longer periods, which is ethically challenging considering the risk of precipitating adrenal crisis. Attempts to minimize the effect of DiCO when studying cognitive function in ACI through standardized study designs will help identify the isolated effect of cortisol deficiency.

#### Attempts to minimize DiCO

Given the deleterious effect of cortisol dysregulation on cognition, it is possible that the DiCO state in ACI drives some of the cognitive dysfunction associated with this disorder. Attempts to better simulate cortisol rhythms have focused on changing dose frequency and using alternative GC formulas or modes of administration (60). However, their effects on cognitive function are understudied.

##### Alternative HC regimens

Studies examining the effect of daily HC regimen on circulating cortisol and ACTH levels in ACI have shown mixed results. Some studies support the use of 3 daily doses (61-63) or either 2 or 3 daily doses (64), while others support the use of 4 (65) or 5 daily doses (61, 63). Moreover, in a cohort of patients enrolled in the European Adrenal Insufficiency Registry, 25 different treatment regimens were used to deliver a daily dose of 20 mg HC (66). The differential effects of all these dosing regimens on cognitive function are unknown.

Specific CAR profiles have been associated with better or worse executive function (45, 67, 68), but attempts to imitate CAR by adjusting night cortisol exposure had no effect. For example, in hospitalized patients with PACI, increasing overnight HC infusion to 0.8 mcg/kg/min at 05:00 to 06:00 and then to 0.9 mcg/kg/min at 06:00 to 07:00 did not improve cognitive function or well-being (69).

##### Alternative GC formulations

Two longer-acting oral HC formulations have been developed to better simulate physiological cortisol rhythms and daily total cortisol exposure (60).

A dual-release oral HC formula (DR-HC, Plenadren^®^), approved for use in Europe in patients with ACI, combines an immediate-release dose to provide a morning cortisol peak and a sustained-release dose to provide a gradual cortisol decrease thereafter, reaching a nadir in the afternoon (70). In one study of 20 patients with either PACI or SACI treated for at least 4 weeks with DR-HC, the authors found significantly better outcomes on assessments of intellectual ability and executive function in patients with PACI than in those with SACI (71). All other domains tested, including attention, short-term memory, and alertness showed no difference, and no significant difference was seen when 18 patients with ACI treated with DR-HC were compared to patients with ACI on conventional HC treatment. Of note, patients treated with the higher dose of DR-HC reported better quality of sleep, but treatment with DR-HC also led to significantly longer time to fall asleep (71). As discussed below, there is a strong association between cognitive impairment and abnormal sleep (72-74). It will be important in future studies to consider a possible effect of sleep disturbance on cognitive function in patients with ACI treated with DR-HC.

A modified-release oral HC formula (Efmody^®^ or Chronocort^®^) is approved for use in Europe in patients with classical congenital adrenal hyperplasia (CAH) (75). When taken at night before bedtime, this formulation begins to release HC after a few hours, reaching peak level in the morning (76). A smaller second dose taken in the morning provides cortisol coverage for the rest of the day (77). The effect of this formulation on cognitive function in patients with ACI remains unknown.

Of note, although prednisolone and its prodrug prednisone can provide longer GC coverage during waking hours (78) given their longer circulating and biological half-lives compared with HC (79), the effects of prednisolone on cognition in ACI remain unexplored.

##### Alternative HC administration routes

As discussed above, administration of HC via subcutaneous infusion can more closely reproduce circadian and possibly ultradian cortisol rhythm. Review of 6 studies evaluating this modality showed fewer hospitalizations for adrenal crisis and improved reports of quality of life (QoL) (80). However, studies that evaluated cognitive function in states of cortisol deficiency, first in healthy males with metyrapone-induced ACI and then in PACI, showed that simulating circadian rhythm alone did not improve cognitive function compared to patients taking conventional oral HC doses (56, 57). Simulating both circadian and ultradian rhythms improved a limited aspect of cognitive function in healthy males with metyrapone-induced ACI (56) but not in PACI (57). As detailed above, multiple factors could have influenced outcomes from this study, including duration cortisol deficiency induced by metyrapone compared with the longstanding deficiency in patients with PACI. A more comprehensive evaluation of cognitive functions in patients treated with this approach is required.

### Inter- and intra-individual variability

Ideally, real-time measures of circulating cortisol levels could be used to understand cortisol's effect on cognitive function. However, such measures are affected by multiple factors that are frequently left unaddressed in studies of patients with ACI, including the well-documented variability in the pharmacokinetics, pharmacodynamics, and tissue-specific responses to oral HC (81). Aging-related cortisol changes, changes in daily HC dose to adjust for stress and sickness, changes in the time of day that HC is taken, and poor adherence further add marked variability. These and other factors limit our ability to effectively monitor adequacy of replacement therapy and to correlate changes in cortisol levels with cognitive function.

#### Absorption, metabolism, and tissue distribution

Total cortisol levels are affected by circulating proteins such as the plasma proteins corticosteroid-binding globulin (CBG) and albumin, which carry approximately 85% of circulating cortisol and are regulated by nutrition, estrogen levels, kidney and liver diseases, and states of critical illness (82-86). All of these factors can influence oral HC absorption, as can concomitant food consumption (59) and HC dose and timing (63, 87).

Body size and fat volume influence the volume of distribution, which affects circulating cortisol levels and HC dosing requirements (59, 88-90). In the liver, the CYP3A4 enzyme converts HC to 6β-hydroxycortisol. CYP3A4 availability, occupancy, and polymorphisms all affect HC availability (91, 92). Renal excretion of cortisol varies with age, sex, and renal function (85). Both renal and liver failure can significantly change cortisol clearance and metabolism (84-86). Additionally, tissue cortisol level, which may not be reflected in circulating cortisol level, is governed by tissue-specific activity of 11β-HSD enzymes, with 11β-HSD1 converting inactive cortisone to active cortisol, and 11β-HSD2 inactivating cortisol back to cortisone (93-95). This conversion also occurs in the brain, likely interfering with our ability to associate circulating cortisol levels with brain function (96, 97).

Circadian rhythms also affect HC pharmacokinetics. For example, an evening dose of HC takes longer to clear from the circulation than does a morning dose (98).

Tissue-specific responses to HC and GR sensitivity are influenced by *NR3C1* polymorphisms, which regulate GR sensitivity (99). The α and β isoforms of the GR and their sub-isoforms generated by alternative translation and post-translational modifications affect GR functionality (100, 101). Variability in chromatin landscape and accessibility of glucocorticoid-responsive elements further influences DNA binding, protein interaction profiles, and downstream cortisol signaling pathways (102, 103).

Where possible, these factors should be taken into consideration when designing a study on cognitive function in patients with ACI.

#### Aging

Changes in HPA axis regulation and circadian rhythm have been reported with aging, largely independent of sex and body size. Studies show a flattening of the circadian rhythm, with smaller slope of decline in cortisol level during the day, higher nadir level at evening and night, as well as reduced intensity of peaks and shortened quiescent periods, attenuated CAR, and decreased sensitivity to GC negative feedback (104). With each decade of age, the lowest cortisol level (quiescent phase) rises by about 0.4 mcg/dL (10 nmol/L); the daily cortisol peak (acrophase, usually in the morning) occurs about 24 minutes earlier (105). Also, ACTH levels were shown to be higher in the eighth decade of life as compared to the third decade (106), and both ACTH and cortisol levels were higher in older subjects while their diurnal amplitude declined (107). Finally, cortisol's negative feedback regulation of ACTH is reduced in older subjects, which results in prolonged exposure to cortisol (108-110).

Cognitive decline observed with aging (111, 112) may be associated with endogenous changes in cortisol levels, although a cause-and-effect relationship between the two is yet unclear. Longitudinal cortisol monitoring of healthy elderly subjects over 3 to 6 years suggests hippocampal aging is involved, as it results in both a decreased capacity for learning and memory consolidation as well as loss of hippocampal inhibition of the HPA axis (113).

Accounting for age as a potential confounder may need consideration when studying cognitive function in patients with ACI.

#### Medication use and adherence

Poor adherence to prescribed regimens may be misinterpreted as response variability. A study of 81 patients with ACI from 5 European countries found that 85% reported some degree of nonadherence to GC replacement therapy. Among these, 35% took their medication at different times than advised, 30% forgot to take it, 11% took less than the prescribed dose, and 4% stopped taking the medication entirely (114). Whether cognitive dysfunction contributes to a lack of adherence in patients with ACI has not been explored. Nevertheless, careful attention to dose adherence is important when considering the effect of cortisol deficiency on cognitive function.

At the same time, the challenges in management of intercurrent illnesses and predicting the development of adrenal crisis can lead to inappropriate use of treatment (115). Examination of diaries from 80 patients with ACI showed increases in daily HC dose in response to symptoms they attributed to low cortisol levels (116). In some cases, however, patients doubled or even tripled their doses despite having mild symptoms and no imminent risk for adrenal crisis. It is possible that fear of adrenal crisis may trigger multiple increases in daily HC administration, and the cumulative effect could be detrimental to cognitive function.

### Excessive GC treatment

There is some evidence that the daily HC treatment doses reported in studies of ACI may have been excessive. As prolonged hypercortisolism is an established risk factor for cognitive dysfunction, the possible effect of overtreatment should be considered when evaluating cognitive function in patients with ACI.

Guidelines recommend HC doses of 15 to 25 mg for patients with PACI (14) or 15 to 20 mg daily for patients with central ACI (13). These recommended doses were calculated in small studies in subjects at rest, based on reports of a cortisol production rate of 5.7 to 7.0 mg/m^2^/day (90, 117, 118), and assumed losses from absorption, hepatic processing, and metabolic bioavailability (119). However, studies have demonstrated that oral HC is easily absorbed by diffusion in the human gut (120), and oral HC bioavailability is at least 95%, with negligible hepatic processing (98, 121). Therefore, the suggestion that higher HC doses may be needed in an attempt to correct for poor bioavailability may in fact lead to overtreatment.

The risk of overtreatment may be increased by residual production of small amounts of endogenous cortisol, particularly in SACI (122). As residual adrenocortical function is not evaluated during HC treatment, its potential contribution to overtreatment may go unnoticed.

Results of studies specifically evaluating cortisol levels in treated patients with ACI indicate that overtreatment is widespread. Measures of serum cortisol and 24-hour urinary free cortisol in 32 patients with ACI showed that 75% required dose reduction to achieve the normal reference range (123). Measures of hair cortisol in 132 patients with ACI showed that 34% had levels above the normal range (124), and salivary cortisol day curves calculated for 20 patients with PACI showed that 18 were overtreated (125).

Not correcting for body size can contribute to the use of overly high HC doses (59). For example, assuming a cortisol production rate of 5.5 to 8.0 mg/m^2^, calculations for a patient with a body surface area (BSA) of 2.7 m^2^ (approximate body mass index [BMI] 50 kg/m^2^) would yield a dose of 14.9 to 21.6 mg HC daily. As only 5 and 10 mg HC tablets are available, this patient would be treated with 15 to 22.5 mg/day. However, the same calculation for a patient with a BSA of 1.6 m^2^ (approximate BMI 18.5 kg/m^2^) would yield a lower dose of 8.8 to 12.8 mg HC daily and treatment with 10 to 15 mg/day.

Yet, correcting for BSA/BMI may not be enough. One group of researchers individually titrated HC dose in 25 patients with ACI based on age, BMI, BSA, blood pressure, and serum sodium levels. The calculated required mean daily dose of 7.6 ± 3.4 mg/m^2^/d (range, 1.9-14.4) was significantly lower than the midpoint dose of 11.8 mg/m^2^ that would have been prescribed using guideline-recommended doses adjusted for BSA only (126). Indeed, the authors suggested that the currently recommended daily HC doses are approximately 30% higher than necessary (127).

These authors also suggest that overtreatment with HC may explain why the cardiovascular profile of patients with ACI is similar to that typically associated with hypercortisolism (127). Indeed, increased cardiovascular mortality has been observed in patients with ACI (128-132), particularly when daily HC doses are increased above 20 mg (131, 133). Furthermore, increased risk for conditions caused by hypercortisolism, such as diabetes mellitus, hyperlipidemia, and hypertension (130-132), have all been reported, as have increased rates of endothelial dysfunction (134) and arterial stiffness index (135).

**Figure 3.**
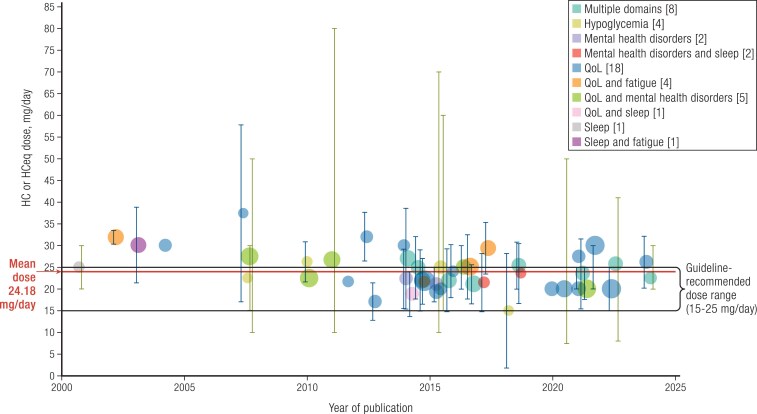
Reported daily HC treatment of patients with ACI. Representation of HC or HC-equivalent doses administered in 46 studies (4, 10, 11, 57, 64, 65, 71, 136-174) examining domains that may be associated with cognitive function in patients with ACI. Number of studies per domain is noted in brackets. Size of each bubble is proportional to the log of the number of subjects. The mean HC-equivalent dose of 24.18 mg/day across all studies and the current guideline-recommended dose range of 15-25 mg/day are indicated. Data are presented as mean and SD (blue bars), median and IQR (blue bars), or median and range (green bars). To identify studies, a PubMed search was conducted with the keywords “*adrenal insufficiency*,” “*adrenocortical insufficiency*,” “*Addison disease*,” “*Addison's disease,*” “*hypopituitarism*,” “*hypopituitary*,” “*hypothalamic-pituitary-adrenal*,” “*HPA axis*,” “*congenital adrenal hyperplasia*,” and “*glucocorticoid*,” “*corticosteroid*,” “*hydrocortisone*,” “*cortisone*,” with either “*quality of life*,” “*cognition*,” “*sleep*,” “*fatigue*,” “*mood*,” “*depression*,” “*anxiety*,” “*hypoglycemia*,” or “*neuroglycopenia.*” A similar search was conducted on Google Scholar to identify any additional articles. Articles were included if published between January 1, 2000, and December 31, 2024, were available in the English language, and studied adult human subjects. Articles were excluded if the population studied did not include patients with ACI or if fewer than 5 patients were studied; if no GC type was provided, if HC or cortisone acetate (CA) was not used at all, or if dose was not given as mg/day; and if the outcome studied did not include one of the domains of interest. If multiple GC types were used, only data on HC/CA were used. For studies that reported mean HC dose overall or by group, the graph shows the reported overall mean or a calculated mean of reported group means. If multiple SDs were provided for multiple groups, the largest SD was used. For studies that did not report mean HC dose, the graph shows reported median and IQR or calculated median and range. For studies that included CA, HC dose equivalents were calculated, with every 1 mg of CA equivalent to 0.8 mg of HC. If GC dose was changed as part of the study intervention, the baseline GC dose was used. Graph was created using JMP (version 17.0.0, SAS Institute, Cary, NC).

Most important, overtreatment is evident in studies examining cognition and factors that can indirectly affect cognition. We surveyed 46 studies published between 2000 and 2024 that evaluated fatigue, mental health disorders, sleep, hypoglycemia, and QoL in a total of 5,166 patients with ACI. As shown in Fig. 3, we found that patients were being treated with a mean HC-equivalent dose of 24.18 mg/day, which is nearly at the upper end of the guideline-recommended range of 15-25 mg/day, with large standard deviations. Importantly, despite the increasing awareness of the risks of overtreatment in ACI, we detected only a minimal decrease in HC daily doses in more recent years.

Careful attention to HC dose levels is important when designing studies of cognitive function assessment in patients with ACI.

### Dose adequacy

A lack of reliable biomarkers challenges our ability to determine and ensure adequate GC replacement. This, in turn, hampers our ability to define the relationship between cortisol level and cognitive function in ACI. Although daily cortisol curves would be preferred over a random daily measure, multiple daily blood draws are time- and effort-consuming for both patients and medical staff and are costly as a routine measure. Testing 24-hour urinary free cortisol is more feasible clinically, but it does not identify fluctuations throughout the day in response to HC treatment (175). Salivary free cortisol can easily be collected multiple times a day by the patient at home, but wide inter-individual variability is seen in patients with ACI, and results correlate poorly with plasma cortisol level (81, 176). As previously reviewed (177), cortisol immunoassays lack specificity and are not standardized. Mass spectrometry that may improve accuracy is expensive and labor-intensive due to the need for extensive validation and is not widely available yet.

Other cortisol-specific biomarkers that have been reported in ACI include salivary cortisone (178, 179), urine cortisone to cortisol ratio (180), and hair cortisol level (124); transcriptional biomarkers such as FKBP5 (181) and miR-124 and miR-375 (182, 183) have also been studied. However, protocols for collection and analysis are not robust, and none have been validated for daily monitoring of HC treatment.

Advanced techniques to continuously monitor cortisol levels and rhythms are being explored to assist in monitoring adequacy of treatment and provide real-time feedback (184). If validated, such approaches would be highly useful in studying the effects of GC replacement therapy on cognitive function in ACI.

## Considering the Effects of ACI-Associated Factors on Cognitive Function

ACI is associated with a wide range of comorbidities, complications, and symptoms that may themselves influence cognitive function (Table 2). However, the extent to which they, and the medications used to manage them, contribute to cognitive performance remains largely unclear.

**Table 2.**
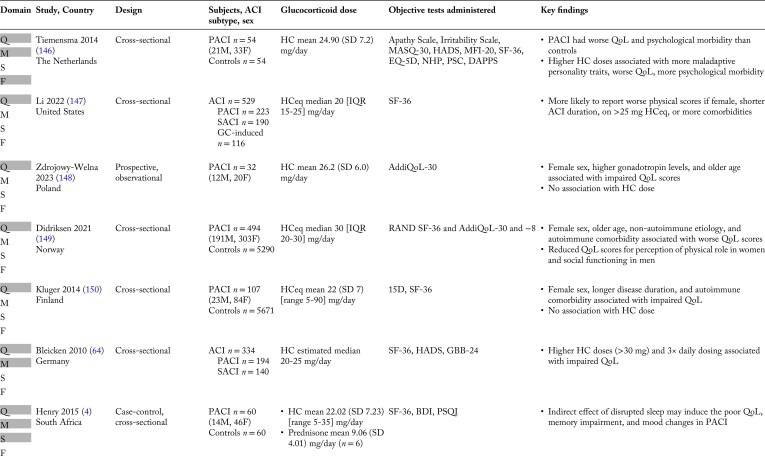

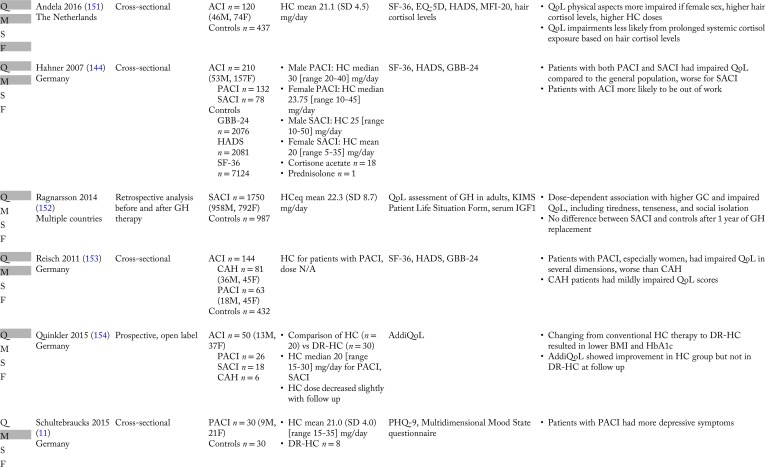

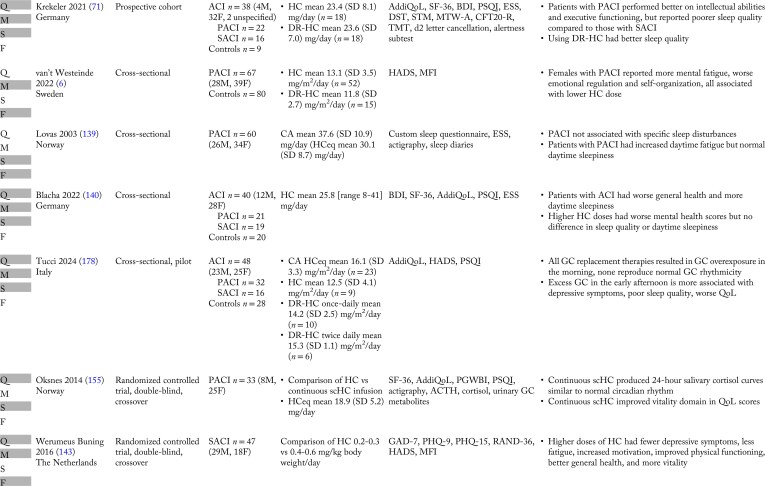

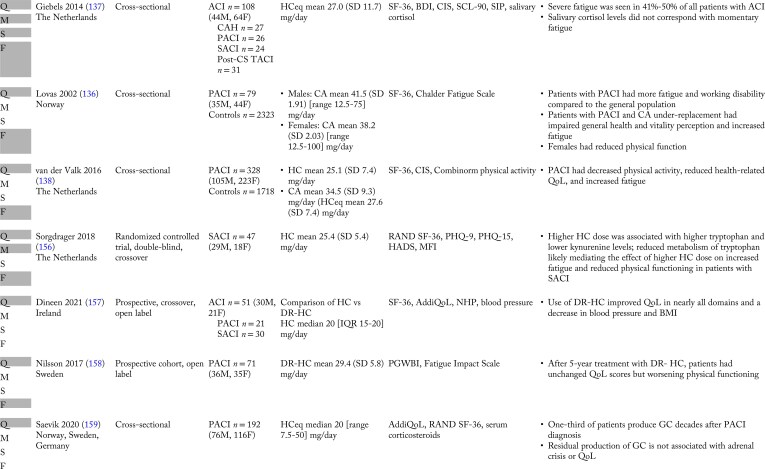

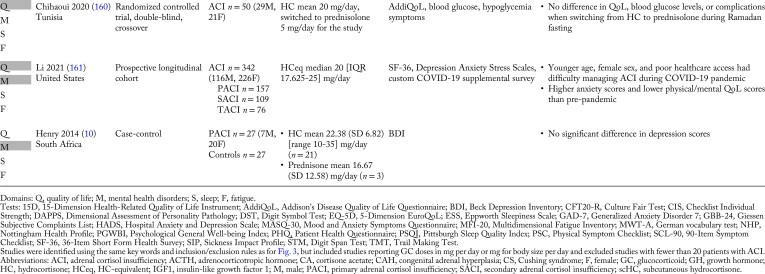
Summary of select studies evaluating quality of life, mental health disorders, sleep quality, and fatigue in adult patients with ACI

## ACI Comorbidities and Complications

### Primary and secondary ACI

Patients with PACI often have accompanying hypoaldosteronism, primary hypothyroidism, and, particularly in women, hypogonadism (185). Rarely, patients may also present with autoimmune polyglandular syndromes involving multiple endocrine glands, as well as other autoimmune conditions such as pernicious anemia (186). The specific contribution of mineralocorticoids to cognitive function is still to be determined (187, 188). Patients with PACI caused by classical CAH are known to experience cognitive dysfunction, potentially due to excessive GC treatment, hyperandrogenism, or a combination of both (189). Prolonged prenatal and childhood exposure to high androgen levels has also been shown to result in lasting cognitive deficits (190).

Adult patients with central ACI often develop comorbid central hypothyroidism, hypogonadism, and GH deficiency (13). A direct relationship between hypothyroidism or hypogonadism and cognitive dysfunction has not been clearly established, as studies show conflicting results. Moreover, hormone replacement did not conclusively show improvement in cognitive functions (191-197). Both thyroid hormone and testosterone exert a profound effect on the brain and, if deficient, would have been expected to cause some cognitive dysfunction. It is possible that the diverse actions of these hormones, heterogeneity of study designs, and variability in endpoints may have contributed to the conflicting results.

More evidence supports a direct relationship between GH deficiency and cognitive dysfunction in adult humans and that GH replacement may help reverse some of these manifestations (198, 199). Yet, many adults with GH deficiency remain untreated due to barriers in diagnosis, access to therapy, adherence, monitoring challenges, and concerns about tumor regrowth (200).

Finally, many patients with central ACI undergo surgery and/or radiation therapy to treat an underlying tumor, which can directly impact normal brain tissue and further influence cognitive outcomes.

### Tertiary ACI

GC inhibits the HPA axis at all levels, suppressing hypothalamic CRH, pituitary ACTH, and adrenal cortisol secretion (201). TACI after endogenous CS or exogenous GC-induced hypercortisolism, is expected to affect cognitive function differently than either PACI or SACI because it is preceded by a long-term hypercortisolism.

After cessation of prolonged GC treatment or cure from CS or MACS, patients frequently remain adrenal insufficient, and recovery of HPA axis function can vary widely. Of 108 patients with adrenal adenoma causing hypercortisolism, 74% of patients with CS and 57% of patients with MACS showed ACI on postoperative ACTH stimulation testing after successful unilateral adrenalectomy. Median time to recover among patients with CS due to adrenal adenoma was 6 months (interquartile range [IQR] 1.4, 17.1), and for patients who had MACS 2.1 months (IQR 0.75, 4.6) (202). In another study of 18 patients with Cushing disease, all showed postoperative ACI but only 12 (67%) had recovered after a median of 24 months (range, 7 months to 4.5 years). The remaining patients had not shown recovery even after 7.5 years (range, 3-12 years) (203).

As briefly reviewed above, a prolonged state of hypercortisolism detrimentally affects brain and cognitive function (7). Reversibility of cognitive dysfunction after resolution of the hypercortisolemic state with cure remains unclear, and the time required to improve is also unknown. Some studies demonstrated short-term recovery on assessments of attention, verbal memory, and processing speed and suggest that further improvement may be seen with time, but others suggest recovery is incomplete (204-208). Importantly, extent of and time to recovery can differ, at least in part depending on duration and severity of hypercortisolism, patient age, comorbidities, and degree of hippocampal damage. Whether clinical or subclinical epigenetic changes driven by hypercortisolism contribute to cognitive dysfunction also remains unclear (209, 210).

Assessing cognitive dysfunction in patients with TACI can be further complicated by the presence of GC withdrawal syndrome (GWS), which is caused by GC dependency (211). GWS is experienced by many patients with ACI after cure of endogenous CS (212) or after stopping long-term, high-dose treatment with GC (213). Symptoms may overlap with those of CS or ACI, as well as with mental health, behavioral, and cognitive disorders. Although the etiology of GWS is unclear, CRH, POMC, vasopressin, central noradrenergic and dopaminergic pathways, prostaglandins, and cytokines were all suggested as mediators (211). Given the difficulties of isolating GWS symptoms from the other pathologies, no studies have directly evaluated cognitive functions in patients with GWS.

## Common ACI Symptoms

Cognitive changes in ACI can be caused or exacerbated by symptoms of ACI. Moreover, medication taken to treat these problems can themselves exacerbate cognitive impairment, especially in older populations (214). Accordingly, their effects on cognition should be taken into consideration in assessments of patients with ACI.

### Fatigue

Fatigue is the most common presenting symptom in ACI, yet its underlying mechanism remain unknown. All untreated patients report fatigue, and it often persists even despite HC replacement therapy (136-138). Among 328 patients with treated PACI, 61% reported fatigue, and 43% described their fatigue as severe. The patients' levels of fatigue were significantly higher than that observed in healthy controls, surpassed those reported by individuals with a history of cancer, and were comparable to those reported by patients who had a history of stroke (138). Increased general and physical fatigue have been reported at similar rates in female and male patients with ACI, whereas greater mental fatigue was reported in women (6, 136).

Since a positive correlation between fatigue and diminished cognition is widely seen in patients with chronic diseases (215), fatigue should be considered a potential confounder when evaluating cognitive function in ACI.

### Sleep disturbances

There is a strong association between cognitive impairment and abnormal sleep (72-74), which is reported at high rates by patients with ACI (139, 216). Accordingly, the possible effect of sleep disturbances on cognition in patients with ACI should be independently considered.

HPA axis activity reaches a nadir at night, remains low for a few hours, and slowly rises in early morning hours, peaking after waking up. In patients with ACI, nadir cortisol levels last through the night. In this setting, increased slow-wave sleep and possibly decreased rapid-eye movement (REM) sleep have been reported (216-218).

Reciprocal effects between sleep-memory interactions and HPA axis activity, specifically CRH and cortisol, are clinically apparent (219). Misalignment of sleep-wake hours such as seen in insomnia and sleep deprivation are associated with HPA axis abnormalities during both sleep and wake hours (217). Moreover, patients with hypercortisolism due to CS demonstrate aberrations in sleep patterns (220, 221), even 1 year after cure (221). Sleep apnea may also be increased in CS (221, 222) and may not improve 1 year after cure (221).

Nevertheless, the extent to which sleep disturbances impact cognition in patients with hypocortisolism due to ACI is unclear. One study showed worse self-reported sleep quality, latency, duration, and efficiency, as well as more sleep medication use and daytime dysfunction in 60 patients with PACI compared with controls; questionnaires of cognitive function also showed worse memory retrieval compared with controls (4). Another study showed worse sleep survey scores and poorer QoL in 40 patients with PACI or SACI compared to controls (140).

However, objective studies did not strongly support patient reports. No clinically significant sleep disturbances were recorded on actigraphy in 60 patients with ACI compared with controls, despite one-third of patients reporting poor sleep quality (139). By contrast, a smaller study of 10 patients showed poor sleep efficiency and more awakenings on actigraphy compared with controls, as well as poorer scores on memory testing that remained unchanged after sleep (141). This same group showed shorter slow-wave sleep in the first half of the night on polysomnography in 7 patients compared with controls, along with worse patient-reported sleep quality and lower sleep scores. However, the results are difficult to interpret, as patients who took their last dose later in the day had abnormally higher residual levels of circulating HC at night than did controls (142).

Based on the currently available data, it is difficult to determine whether self-reported abnormal sleep detrimentally affects cognition in patients with ACI, and no direct cause and effect between reduced quality of sleep and cognitive dysfunction has yet been demonstrated. It is likely that the DiCO state, disturbed cortisol rhythms and GC overtreatment, as well as ACI-associated comorbidities interfere with the ability to isolate sleep's contribution to cognitive function in this population.

### Depression and anxiety

Patients with PACI and SACI frequently report depression, which itself is an established risk factor for cognitive impairment (223). One study involving 58 patients with PACI found higher depression scores compared to 60 matched controls (4). Another study showed that 6.5% of 278 patients with PACI experienced depression (224). In a separate randomized, double-blind, crossover study, 63 patients with SACI reported symptoms of depression while receiving a daily HC dose of 15 to 20 mg. These symptoms significantly improved when the daily dose was increased to 30 to 40 mg, prompting the authors to consider that HC doses higher than commonly accepted may be necessary when managing patients with SACI and concomitant symptoms of depression (143).

Neuropsychological assessment of 67 young patients with autoimmune PACI and 80 controls showed higher levels of anxiety in the PACI cohort, which was linked to both increased executive dysfunction and fatigue (6). Notably, the presence of executive dysfunction was more prominent in female patients. Those with self-reported executive dysfunction related to self-organization and mental fatigue were also at a higher risk of developing depression. Furthermore, 20% of patients, particularly those who were female, reported experiencing difficulties with emotional regulation, defined as a reduced ability to recover mentally and calm down after experiencing a stressful emotion. Higher doses of HC were associated with improved emotional regulation but not anxiety, leaving the authors to suggest that GC dose optimization may be necessary for management of these patients (6).

Depression and anxiety in ACI may be, at least in part, a function of the chronicity of disease, as similar anxiety levels were demonstrated in patients treated for PACI and those with well-controlled type 2 diabetes mellitus (225). Nevertheless, in a study of 256 patients with ACI, significantly worse scores on self-reported anxiety questionnaires were seen in both PACI and SACI compared with normative data even after excluding respondents with concomitant nonendocrine or endocrine disease, and depression scores were worse in the SACI cohort (144).

The frequency of depression and anxiety reported by patients with ACI suggests a need to account for their presence as a potential confounder when assessing cognitive function in patients with ACI.

### Hypoglycemia

Hypoglycemia can be seen in adult patients with untreated ACI, especially during an adrenal crisis (226-228). Hypoglycemia is commonly described in children with PACI (229, 230). Recurrent hypoglycemic events in patients with diabetes mellitus accelerates cognitive dysfunction and increases risk for dementia (226, 231-234). Given the connection between cognitive dysfunction and glucose metabolism variability (235, 236), the possibility that hypoglycemia influences reported cognitive dysfunction in patients with ACI should be considered.

Cortisol has a dominant effect on glucose metabolism and is a major counterregulatory hormone of insulin. In patients with ACI treated with HC, the cortisol/insulin regulatory imbalance resulting from the DiCO state can potentially predispose patients to hypoglycemia, mostly in the second half of the night, when cortisol levels do not gradually rise as expected.

There is some evidence to suggest that patients with ACI experience nocturnal hypoglycemia. Continuous glucose monitoring (CGM) demonstrated nocturnal glucose < 50 mg/dL in 2 patients with PACI without diabetes who reported disturbed sleep, morning headaches, and fatigue despite HC replacement, and symptoms resolved after changes in HC timing and nutrition (237, 238). Similarly, among 6 patients with SACI, CGM demonstrated nocturnal fasting glucose levels < 70 mg/dL in 5 of 6 patients with morning headaches and general discomfort, which were reversed after changes to HC treatment time (145). If indeed hypoglycemia is present in patients with ACI despite treatment, this could further confound assessment of cognitive function in patients with ACI.

## Cognitive Screening Tests and Symptom Surveys in ACI

Early detection of cognitive dysfunction can enable timely and appropriate treatment and support, monitor brain health and disease status, reduce functional disability, and improve day-to-day life engagement (239-241). Prospective, longitudinal, controlled studies will capture dynamic cognitive and behavioral changes over time and help define impairments specific to ACI.

### Cognitive Screening Tests

Feasibility, accessibility, and psychometric quality of cognitive tests should guide instrument selection within the individual clinical context. Considerations such as time constraints within the clinical setting and test format may also contribute to frontline decision-making to best meet the identified clinical need (Table 3). Standardized cognitive screening tools, such as the Montreal Cognitive Assessment (MoCA) (248), St. Louis University Mental Status Exam (SLUMS) (255), the Addenbrooke Cognitive Examination (ACE-III) (242), and the Repeatable Battery for the Assessment of Neuropsychological Status (RBANS) (252-254) assess the 5 core cognitive domains of attention/concentration, language, memory, visuospatial skills, and executive function. The Mini-Addenbrooke's Cognitive Examination (Mini-ACE) (246) and Mini-Cog (247) are very brief cognitive screening measures; the Mini-ACE assesses orientation, memory, verbal fluency, and visuospatial skills, while the Mini-Cog assesses memory and executive function.

**Table 3.** Select cognitive screening tests

	Domains assessed						Approach		Duration (mins)
Cognitive test	Orientation	Attention	Executive function	Memory	Fluency/Language	Visuospatial function	Objective	Subjective	
ACE-III*^a^*	√	√	√	√	√	√	√		15-20
Mini-ACE*^a^*	√	√		√	√	√	√		5
Mini-Cog*^a^*			√	√			√		5
MoCA*^a^*	√	√	√	√	√	√	√		15
RBANS*^a^*		√	√	√	√	√	√		20-30
SLUMS*^a^*	√	√	√	√	√	√	√		10
BTACT*^b^*		√	√	√			√		15
TICS*^b^*	√	√		√	√		√		10-15
NIHTB-CB*^c^*		√	√	√	√		√		30
BOCA*^c^*	√	√	√	√	√	√	√		15
C3B*^c^*		√		√			√		10
MTMCF*^c^*		√	√	√	√		√		30
PROMIS-CF*^d^*		√	√	√				√	2-5
PROMIS-CFA*^d^*		√	√	√				√	2-5

Abbreviations: ACE-III, Addenbrooke's Cognitive Examination III (242); BOCA, Boston Cognitive Assessment (243); BTACT, Brief Test of Adult Cognition by Telephone (244); C3B, Cleveland Clinic Cognitive Battery (245); Mini-ACE, Mini-Addenbrooke's Cognitive Examination (246); Mini-Cog (247); MoCA, Montreal Cognitive Assessment (248); MTMCF, The Mobile Toolbox for Monitoring Cognitive Functioning (249); NIHTB-CB, NIH Toolbox–Cognitive Battery (250); PROMIS-CF, PROMIS v2.0 Cognitive Function–Short Form (251); PROMIS-CFA, PROMIS v2.0 Cognitive Function Abilities–Short Form (251); RBANS, Repeatable Battery for the Assessment of Neuropsychological Status (252-254); SLUMS, St. Louis University Mental Status Exam (255); TICS, Telephone Interview for Cognitive Status (256-258).

^*a*^Paper and pencil test to be administered in clinic by a provider.

^*b*^Telephonic test, permitting geographical flexibility; efficiency and scalability.

^*c*^Digital, cognitive screening tools with consistent, efficient administration and automated scoring.

^*d*^Subjective, self-reported cognitive status.

Telephone assessments allow for cognitive screening when in-person visits are not feasible. For example, the Telephone Interview for Cognitive Status (TICS) (257, 258) assesses orientation, attention, memory, and verbal fluency. The Brief Test of Adult Cognition by Telephone (BTACT) (244) measures memory, executive functioning, reasoning, and processing speed and has been used to assess cognitive impairment in patients with PACI (10).

Efficient and reliable digital neuropsychological assessment measures are increasingly being adopted (259). Digital platforms for cognitive assessments facilitate ease of administration, have a short administration time, and provide useful data to aid clinical decision-making. For example, the National Institutes of Health Toolbox–Cognitive Battery is a state-of-the-art digital platform for objective cognitive assessment in research and clinical practice (250, 260). The platform includes discrete subtests designed to evaluate attention, processing speed, executive function, learning and memory, and language, and it produces crystalized and fluid intelligence composite scores (250). The Boston Cognitive Assessment (BOCA) (243), the Cleveland Clinic Cognitive Battery (C3B) (245), and the Mobile Toolbox for Monitoring Cognitive Functioning (249) are digital, self-administered cognitive screening tools that provide a measure of global cognition and a multi-domain cognitive profile.

Patient-reported outcome (PRO) measures, such as the Patient-Reported Outcomes Measurement Information System (PROMIS^TM^) Applied Cognition instruments, are short assessments of cognitive function that add key information about perceived cognitive status and the impact of cognitive functioning on daily life (251, 261, 262).

#### Key considerations

There is, as yet, no standardization in the use of the numerous cognitive measurements available to assess diseases that affect cognition. More specifically, there is no uniformity on test administration practices, scope of cognitive domains tested, or test selection to assess a particular domain. Strategy for assessing severity of deficits and prognosis also depends on cohort specific contexts and the normative or comparative sample, which can differ by region and culture (262-265).

Furthermore, when assessing executive function and memory, the test environment may inadvertently lead to under- or over-estimating cognitive capabilities (266). A structured testing setting as in a clinic may not capture the functional challenges patients see in conducting day-to-day activities within the home, community, and workplace. By contrast, in decentralized studies, assessments are performed outside of the clinic, potentially providing more real-world measures. In both settings, there can be biases that influence results and contribute to differences in subjective and objective reports.

Careful attention to the psychometric properties of the cognitive assessment tool is also important. Sensitivity, specificity, positive predictive value, and negative predictive value can vary widely due to test construction, clinical setting, normative samples, and patient characteristics. For example, in a patient population with subtle cognitive impairments, as in ACI, tests with higher sensitivity may be preferred to reduce false negatives and to guide referrals to cognitive specialists (263, 264, 267).

Collaborating with a psychologist on selection of a screening measure is critical. For example, the Mini-Mental State Examination (MMSE) (268), historically one of the most frequently used cognitive screening instruments, would not be a good cognitive screening tool for patients with ACI. The MMSE has limited ability to assess memory and executive functioning, for example, and lacks adequate sensitivity to identify subtle or mild cognitive difficulties. Good clinical practice in cognitive screening necessitates knowing the boundaries of one's knowledge and practice competencies, the value of and limitations to cognitive screening tools, and use of subjective and objective data to guide appropriate referrals for more thorough workups by the respective specialists in the area. Such referrals can then use multimodal, multidimensional, and multi-method assessments to disentangle competing contributing factors, maximize validity of clinical judgment, and guide clinical care pathways (263).

Ideally, a comprehensive neuropsychological battery should evaluate the following elements: (1) general intellect; (2) academic skills (ie, reading; writing; arithmetic); (3) attention, concentration, and working memory; (4) processing speed; (5) executive functioning (ie, set-shifting, problem solving, reasoning, and metacognition); (6) memory (verbal and visual); (7) language (expressive and receptive); (8) visuo-perceptual skills; (9) sensory-motor function; (10) psychiatric status; (11) social-emotional functioning; (12) pragmatic communication; and (13) adaptive functioning (267).

A formal neuropsychological assessment with standardized, validated tests can also assist with differential diagnosis of brain-based functional impairments (269). For example, patterns of dysfunction might help tease apart whether a memory problem is due to an underlying medical (eg, hyponatremia), neurodegenerative (eg, Alzheimer disease), or mental health condition (eg, depression). This may be particularly useful in patients with ACI, as cognitive decrements in these patients may be subtle and therefore difficult to ascribe to the underlying disease or to a comorbid condition or complication. For example, vascular disease (270, 271), uncontrolled diabetes mellitus (272), and sleep apnea (273, 274) have all been associated with cognitive decline. Furthermore, the establishment of a cognitive baseline is important, as it can help determine whether poorer scores indicate decline or are an artifact of an unrelated cause, such as a pre-existing congenital condition or a low level of education (269). Careful physical and neuropsychological assessment can identify or rule out such confounders in patients with ACI.

### Symptom Surveys

Patient-reported questionnaires of ACI-associated symptoms are an important complement to the cognitive screen given their strong correlation with cognitive dysfunction (275-277) (Table 4). Validated screens for anxiety, including the Beck Anxiety Inventory (278), PROMIS-Anxiety Short Form–7a (279), Generalized Anxiety Disorder–7 (GAD-7) (280), and Hospital Anxiety and Depression Scale (HADS) (281), as well as screens for depression, including the Patient Health Questionnaire–9 (282), Beck Depression Inventory (BDI-II) (283), and PROMIS-Depression Short Form–8a (279) each takes only 5 minutes and can be easily administered. Screens for fatigue, such as PROMIS Fatigue-Short Form–7a (284), Fatigue Severity Scale (285), Fatigue Assessment Scale (286), and Chalder Fatigue Scale (287), are similarly brief. Sleep assessment, such as with the Pittsburgh Sleep Quality Index (288) and the Jenkins Sleep Scale (289), is particularly important. Sleep is critical for memory consolidation, and the circadian rhythm disruption and HPA axis inhibition in patients with ACI likely disturbs processes important for consolidation and memory performance (216). Frequent awakening also contributes to poor frontal executive dysfunction and memory difficulties (290). Where possible, objective measures of sleep quality using actigraphy could be employed to capture sleep irregularities in the home setting (291). As interactions among anxiety, depression, and sleep disruption or poor sleep quality may contribute to poor cognitive performance (292, 293), thorough evaluation can help distinguish the effects of each contributing factor.

**Table 4.** Select patient-reported symptom surveys

		Items	Duration (min)
Anxiety	Beck Anxiety Inventory (278)	21	5
	PROMIS-Anxiety Short Form 7a (279)	7	2
	Generalized Anxiety Disorder 7 (280)	7	2
	Hospital Anxiety and Depression Scale (281)	14	4
Depression	Patient Health Questionnaire 9 (282)	9	5
	Beck Depression Inventory II (283)	21	5
	PROMIS–Depression Short Form 8a (279)	8	2
Fatigue	PROMIS–Fatigue-Short Form 7a (284)	7	2
	Fatigue Severity Scale (285)	9	3
	Fatigue Assessment Scale (286)	10	3
	Chalder Fatigue Scale (287)	11	5
Sleep	Pittsburgh Sleep Quality Index (288)	19	8
	Jenkins Sleep Scale (289)	4	2

#### Key considerations

Small cohort size is common in studies of ACI due to the rarity of the disease and can make it challenging to parse small differences between patients and controls. Unfortunately, small cohort size can also contribute to under-identification of health disorders often seen in conjunction with ACI, such as depression, anxiety, and fatigue. Heterogeneity of stringency across research study protocols for allowance of medications that alter mental health and sleep, such as hypnotics, benzodiazepines, and anticholinergics, further complicates an ability to draw firm conclusions.

Use of appropriately targeted surveys in addition to comprehensive cognitive testing would further increase validity of study outcomes and applicability to the clinic. Larger studies with strict protocols should balance cohorts for sex, age, medical comorbidities, and concurrent medications, particularly those that may negatively impact cognitive function, mental health, and sleep.

### Neuroimaging Techniques

Longitudinal volumetric brain imaging is frequently employed in studies of neurodegenerative diseases to follow affected and unaffected at-risk people over time. The goal of these studies is to identify brain structures that may be more vulnerable and thus serve as a biomarker of disease diagnosis and progression (294, 295).

Functional MRI (fMRI) measures blood oxygen level-dependent (BOLD) signals while a person is at rest or participating in a cognitive task. Such a tool may be useful as an adjunct to structural MRI in elucidating whether various networks implicated in attention, vigilance, or working memory are affected (296-298). For example, structural MRI showed reduction in brain volume in patients with active CS compared to healthy controls with only partial reversal after remission, suggesting cortisol has a marked and sustained effect on brain structure (40, 299, 300). On fMRI, patients with Cushing disease demonstrated changes in brain function and connectivity in regions involving cognitive processing (41).

Structural MRI has similarly been used to identify brain changes in patients with ACI. A decrease in total brain volume and intracranial volume of approximately 4% was seen when comparing 52 patients with autoimmune PACI with 70 healthy controls who had similar levels of education and similar scores on depression and anxiety surveys (301). Changes were particularly observed in the inferior and superior parietal cortex, supramarginal gyrus, orbitofrontal cortex, para-hippocampal gyri, and bilateral thalamus. The authors speculate that these areas have a high GR density, which may have contributed to susceptibility of these regions. Indeed, volume decrease correlated with GC dose in areas such as the left lingual gyrus, left anterior cingulate cortex, and right supramarginal gyrus (301). The researchers further demonstrated that patients with ACI exhibit higher resting-state brain functional connectivity in the bilateral orbitofrontal cortex, left medial visual network, and posterior default mode network. These changes were associated with higher GC doses but not with mental fatigue or executive functioning deficits (302). In another study using fMRI, patients with ACI receiving oral HC replacement (20 mg daily, divided into 3 doses) showed higher BOLD signals in regions of the default mode network, specifically the posterior cingulate cortex and precuneus, compared to patients on subcutaenous HC infusion, which more closely reproduces both circadian and ultradian rhythms (57). While MRI findings are promising, their relevance to the underlying pathophysiology of ACI and their relationship with cognitive function remain unclear and warrant further investigation.

Positron emission computed tomography, which uses specialized radiolabeled tracers targeted to specific receptors, has been evaluated in neurodegenerative diseases (303). Radiolabeled GC cannot cross the blood-brain barrier, are metabolically unstable, and are likely too nonspecific to be clinically useful (304, 305). A high-affinity, high-specificity analogue has been reported to bind GR in a rodent model (306). Such a technique, if successful, could be used to visualize GR expression level in the brain, and verify the inverted U-shaped relationship between cognitive function and cortisol levels (44, 45).

## Summary

Studying cognitive function in patients with chronic ACI presents numerous challenges and limitations. Recognizing and addressing patient-reported cognitive dysfunction in the clinic is a crucial first step. A deeper understanding of this symptom requires well-designed studies that account for the complexities and gaps in ACI research, along with the standardization of study designs. Table 5 outlines key challenges and potential approaches to studying cognition in ACI, many of which are also applicable to studies of other comorbid symptoms and conditions in patients with ACI.

**Table 5.** Key challenges and potential solutions to consider when designing a study on cognitive function in ACI

**Overall Study Design**
1. Increase rigor and sample size by performing large, multicenter prospective studies
2. Study patients longitudinally to capture dynamic changes in cognition over time
3. Consider crossover study designs to compare effect of different treatment regimens on cognition
**Study Characteristics**
1. Confirm diagnosis of ACI per guidelines and updated, assay-specific cortisol thresholds
2. Distinguish between GC-induced TACI and other ACI causes
3. Analyze separately PACI and SACI
4. Leverage the expertise of healthcare professionals across disciplines
5. Use validated patient-reported questionnaires and objective tests
6. Account for clinic-based vs decentralized, at-home measurements
**Effects of DiCO and Abnormal Cortisol Rhythms**
1. Standardize treatment frequency and timing •Consider switching to longer-acting GC formulations or subcutaneous HC infusion in ACI-appropriate doses •Use wearables for activity and sleep monitoring to align treatment with the sleep-wake cycle •Assess HC-related diurnal cortisol patterns and CAR •Monitor GC treatment timing
2. Recognize possible confounders •Account for whether HC is taken with or without food •Consider the timing of cognitive tests relative to HC dosing •Consider residual adrenal gland cortisol production •Monitor medication adherence •Tightly match subjects and controls for age, especially in older adults (>60 years)
**Effects of Excessive GC Treatment**
1. Monitor for clinical signs of ACI and overtreatment
2. Account for increased HC doses during adrenal crises or symptomatic periods
3. Adjust HC dose to body size
4. Consider the impact of medications or comorbidities that alter cortisol metabolism
**ACI-Associated Confounders**
1. Account for comorbidities such as untreated hypogonadism or GH deficiency
2. Exclude patients with unrelated neurocognitive diseases
3. Consider the effects of sleep, fatigue, depression, and anxiety
4. Consider the contribution of medications that influence cognition

Abbreviations: ACI, adrenal cortisol insufficiency; ACTH, adrenocorticotropic hormone; CAR, cortisol awakening response; DiCO, dissonant cortisol oscillations; GC, glucocorticoid; GH, growth hormone; HC, hydrocortisone; HPA, hypothalamic-pituitary-adrenal; PACI, primary adrenal cortisol insufficiency; TACI, tertiary adrenal cortisol insufficiency.

One of the major obstacles in studying ACI is its rarity and heterogeneity. Recruiting large cohorts and ensuring appropriate matching for variables that may affect cognition can be difficult. Multicenter studies are preferred as they offer an opportunity for larger sample sizes and allow for better stratification of ACI subtypes. Where only smaller cohorts are available, longitudinal studies could be conducted to assess cognitive function over time and identify risk factors for cognitive decline as compared to controls. Furthermore, randomized crossover studies could compare cognition under different HC regimens in the same individual, minimizing the need for larger cohorts and well as potential confounders from inter-individual variability.

Healthy controls should be well-matched for sex, age, and education, and where possible comorbidities and symptomatology. Including a control group comprising patients with another chronic endocrine disorder such as hypothyroidism or hypopituitarism without ACI can further help isolate ACI-specific effects. Blinding both investigators and participants, particularly in crossover designs, reduces placebo/nocebo effects and minimizes expectation bias.

Digital tools and remote monitoring could improve data collection and patient compliance. For example, wearables that assess heart rate variability and activity level; polysomnography or actigraphy for objective assessment of sleep architecture; and imaging technologies such as fMRI studies that assess brain functional connectivity or diffusion tensor imaging that assess microstructural white matter integrity can all be added to better characterize the disorder's effects. Finally, machine learning models can be developed to identify subtle cognitive patterns predictive of ACI-related dysfunction.

Use of validated screening tests can identify, quantify, and confirm the presence of cognitive dysfunction, including difficulties with executive function and memory. Facilitation of a coordinated approach and leveraging the expertise of healthcare professionals across disciplines will enable a more comprehensive study of patient outcomes and their impact on day-to-day functional engagement and quality of life.

By encouraging discussions in the scientific community on implementing better designed, more rigorous studies, we seek to help bridge gaps in the understanding of cognitive dysfunction in ACI. Clinicians will be better equipped to provide appropriate referrals to neurology, neuropsychology, psychology, psychiatry, sleep medicine, and cognitive rehabilitation as needed. In this way, the knowledge gained from rigorous study of cognitive function in ACI will enable us to better assess and manage our patients in everyday clinical practice.

## Abbreviations

11β-HSD: 11β-hydroxysteroid dehydrogenase
ACI: adrenal cortisol insufficiency
ACTH: adrenocorticotropic hormone
BMI: body mass index
BSA: body surface area
CAR: cortisol awakening response
CRH: corticotropic releasing hormone
CS: Cushing syndrome
DiCO: dissonant circulating cortisol oscillations
DR-HC: dual-release hydrocortisone
fMRI: functional magnetic resonance imaging
GC: glucocorticoid
GH: growth hormone
GR, glucocorticoid receptor; GWS: glucocorticoid withdrawal syndrome
HC: hydrocortisone
HPA: hypothalamic-pituitary-adrenal
IQR: interquartile range
MACS: mild autonomous cortisol secretion
MR, mineralocorticoid receptor; MRI: magnetic resonance imaging
PACI: primary adrenal cortisol insufficiency
QoL: quality of life
SACI: secondary adrenal cortisol insufficiency
TACI: tertiary adrenal cortisol insufficiency
